# Microwave Sensors for Breast Cancer Detection

**DOI:** 10.3390/s18020655

**Published:** 2018-02-23

**Authors:** Lulu Wang

**Affiliations:** 1 Department of Biomedical Engineering, School of Instrument Science and Opto-Electronics Engineering, Hefei University of Technology, Hefei 230009, China; luluwang2015@hfut.edu.cn; 2 Institute of Biomedical Technologies, Auckland University of Technology, Auckland 1142, New Zealand

**Keywords:** lab-on-a-chip, label-free, breast cancer, microwave sensor, microwave imaging

## Abstract

Breast cancer is the leading cause of death among females, early diagnostic methods with suitable treatments improve the 5-year survival rates significantly. Microwave breast imaging has been reported as the most potential to become the alternative or additional tool to the current gold standard X-ray mammography for detecting breast cancer. The microwave breast image quality is affected by the microwave sensor, sensor array, the number of sensors in the array and the size of the sensor. In fact, microwave sensor array and sensor play an important role in the microwave breast imaging system. Numerous microwave biosensors have been developed for biomedical applications, with particular focus on breast tumor detection. Compared to the conventional medical imaging and biosensor techniques, these microwave sensors not only enable better cancer detection and improve the image resolution, but also provide attractive features such as label-free detection. This paper aims to provide an overview of recent important achievements in microwave sensors for biomedical imaging applications, with particular focus on breast cancer detection. The electric properties of biological tissues at microwave spectrum, microwave imaging approaches, microwave biosensors, current challenges and future works are also discussed in the manuscript.

## 1. Introduction

Cancer is a major public health issue worldwide and the second leading cause of death in the United States [1]. In 2017, 1,688,780 new cancers and 600,920 cancer deaths occur in the United States [1]. To date, there is no clear reason why people get cancer, but some factors may increase cancer risk, such as tobacco, obesity, lack of physical activity, alcohol, infections, genetic and molecular mechanisms [2]. Clinical studies showed that early diagnostic methods with suitable treatments improve the 5-year survival rates significantly [3]. Lung cancer, breast cancer, prostate cancer, liver cancer, colorectal cancer and stomach cancer are the most common types of cancers [4], and breast cancer is the leading cause of cancer death among females in the United State [5]. 

Biopsy-based methods are normally used to identify cancerous tissue and benign tissue [6], however, such techniques suffer from high cost and require trained people [7]. Over the past decades, biosensor-based methods to detect cancer have attracted the interest of many people [8,9,10,11,12,13]. A biosensor normally contains target cancer marker, bioreceptor and compatible biotransducer components that play an imperative role and decide the technical specifications of the biosensor device. More recent studies have investigated Lab-on-chip based biosensors for cancer detection due to their high selectivity, sensitivity and specificity [14,15,16]. Additionally, they have the advantages in biological sample processing, high throughput, low reagent and sample consumption, short assay time, and multiplexed detection [17]. The development of lab-on-chip sensor was envisioned to be a useful tool for better understanding of cancer metastasis. It is urgently needed to develop a high sensitivity and label-free method for early rapid diagnosis of breast cancer. A number of biomarker-based methods have been studied for breast cancer detection [18,19,20,21], including radioimmunoassay, immunohistochemistry, enzyme-linked immunosorbent assay, and fluoroimmunoassay. However, the biomarker-based methods have some disadvantages include expensive, time-consuming, require complex labeling process and trained people, and often limited in detection sensitivity [22]. To date, cancer biomarker discovery is still in its discovery stage and the evidence is too restricted to apply biomarker-based methods as the diagnostic tools for early breast cancer detection [23]. For example, protein biomarkers cannot be used as individual biomarkers for identifying of breast cancer as a single marker does not provide sufficient information to confirm the cancer type, and the obtained information is related to the stage of cancer, treatment and the state of subjection.

Apart from biomarker-based methods, screening-based diagnostic techniques are the commonly used methods in hospitals to identify diseases such as breast cancer [24]. For early diagnosis of breast cancer, scientists worldwide have extensively investigated many imaging-based diagnostic methods, including magnetic resonance imaging [25], positron emission tomography [26], mammography [27] and contrast-enhanced digital mammography [28]. However, these screening-based methods are expensive and provide limited image resolution. Although mammography has been considered as the current standard breast imaging tool, it is less effective for dense breasts and small tumors [29,30]. Recently, microwave breast imaging has been proposed as an alternative or additional detection method to mammography for early diagnosis of breast tumor [31]. The microwave breast image quality is affected by the microwave sensors, the synthetic aperture, and the bandwidth of the probing signal [32]. In fact, the rapid growth of microwave breast imaging systems requires high-performance broadband sensor that suitable to detect small tumors with cost-effective, compact and easy-to-use measurement system. 

Typical characteristics of microwave sensor to be applied for breast tumor detection are: wide impedance bandwidth, small size, repeatable and cost-effective fabrication, and ability to efficiently couple power to the breast. Many efforts are underway to identify new sensor characteristics suitable to satisfy the challenging requirements of the microwave breast imaging systems. This paper presents a comprehensive review of the scientific literature of the last decade to provide investigators a valuable support tool to the microwave biosensors for early diagnosis of breast tumor. The present review focuses on three major parts: electrical properties of tissue, microwave breast imaging and microwave sensors. A comparative sensing performance, present challenges, and future prospects of label-free microwave biosensors also discussed in detail.

## 2. Electrical Properties of Tissue 

The microwave imaging based approaches to detect cancer cells are highly related to the dielectric properties contrast between the healthy tissue and the malignant tissue [33]. Different water-content biological tissues have distinct electrical properties [34]. Foster et al. [35] reported a critical review of human tissues. Up to date, scientists have investigated many types of tissues, including breast tissue [36], liver [37], lymph nodes [38], skin [39], bone [40], and heart [41]. Some factors have been reported to explain the difference in electrical properties between healthy and malignant tissues include water content [42], necrosis and inflammation causing breakdown of the cell membrane [43], sodium content [44], charging of the cell membrane [44], and change in the dielectric relaxation time [45].

### 2.1. Dielectric Properties of Breast Tissues 

Joines et al. [46] measured various fresh tissues (include colon, kidney, liver, breast, muscle and lung) and malignant tissues from patients at frequency range of 50~900 MHz. It was found that the conductivity contrast between malignant tissue and normal breast tissue is 6.4:1 and the relative permittivity contrast between malignant tissue and healthy breast tissue is 3.8:1. For the same type of tissue, the dielectric contrast between malignant and normal tissues is greatest for the mammary gland. Jin et al. [47] reported that the dielectric contrast between healthy breast tissue and malignant breast tissue appears to be inhomogeneous due to the dielectric property changes. 

Gabriel et al. [48,49,50] extensively measured the dielectric properties of 30 different tissues and found that the dielectric properties of muscle or malignant tumors (high-water-content) are higher than fat or normal breast tissues (low-water-content) over the entire radio frequency (RF) spectrum of power frequencies through millimeter waves. Lazebnik et al. [51] studied the dielectric properties of normal breast tissue and cancerous tissue at frequency range 0.5~20 GHz. Their research findings illustrated that both dielectric constant and conductivity decrease with the adipose content increase, and conversely as the percentage of glandular and/or fibro-connective tissue increase, both dielectric constant and conductivity increase. The dielectric properties of normal breast tissues having a wide range of values depending on tissue type. Chaudhary et al. [52] measured the relative permittivity and conductivity of normal and malignant breast tissues at RF and microwave frequencies (up to 3 GHz). The dielectric contrasts between malignant tissue and normal breast tissue were, 4.7:1 and 5:1, these results were in good agreement with Joines et al.’s results.

The dielectric properties of malignant tumors at different frequencies have been measured and investigated by many research groups. The experimental results showed that the relative permittivity and conductivity of high-water-content tissues are almost the same as muscle at frequencies above 1 GHz, however, the relative permittivity and conductivity of malignant tumors are significantly higher than a muscle at frequencies below 1 GHz [53]. Swarup et al. [54] studied the dielectric properties of MCA1 fibrosarcoma mouse tumors at different days after inception. No significant variations of relative permittivity and conductivity were seen by tumor age. While the larger tumors exhibited a necrotic interior, they showed little difference in the relative permittivity and conductivity above 0.5 GHz. Surowiec et al. [55] investigated cm-size human breast malignant tissues and adjacent tissues. The dielectric properties of normal breast tissue increased as malignant tissue. This effect may be caused by infiltration or vascularization. It could enlarge the microwave scattering cross-section and thereby aid in the confocal microwave detection of the tumor.

### 2.2. Modelling of Biological Tissue

The dielectric properties of biological tissues are nonlinear functions of frequency [56]. Therefore, it is a critical task to choose a suitable working frequency for microwave breast imaging system due to the attenuation of microwave signal increases with the frequency and increase in the conductivity resulting in a lower penetration depth. Various numerical models include the most popular Debye and Cole-Cole models have been developed for modelling the biological tissues [57]. The Debye model is defined as follows [45]:

(1) $$\varepsilon_{r}=\varepsilon_{\infty }+\frac{\varepsilon_{s}+\varepsilon_{\infty }}{1+j\omega \tau }-j\frac{\sigma }{\omega \varepsilon_{0}}$$

where $\varepsilon_{\infty }$ is the permittivity and its value highly relative to the water content of the tissue, $\varepsilon_{s}$ is the static permittivity, τ is the relaxation time. The measurements from Joines et al. and Chaudhary et al. were extrapolated to higher frequencies using the Debye model. 

The Cole-Cole model was developed to represent the complex dielectric constant [58]:

(2) $$\varepsilon^{*}\left(\omega \right)=\varepsilon_{\infty }+\frac{\varepsilon_{s}-\varepsilon_{\infty }}{1+{\left(j\omega \tau \right)}^{1-\alpha }}$$

where $\varepsilon^{*}$ is the complex dielectric constant, $\varepsilon_{s}$ and $\varepsilon_{\infty }$ are the static and infinite frequency dielectric constants, ω is the angular frequency and τ is the time constant. The exponent parameter α, which takes a value between 0 and 1 describes different spectral shapes. When α=0, the Cole-Cole model reduces to the Debye model. When α>0, the relaxation is stretched. 

Zastrow et al. [59] applied the empirical relationship between the dielectric parameters and the moisture content model (developed by Foster and Schwan) to further confirm the Debye model. For an approximate 10% moisture content, the empirical model is:

(3) $$\varepsilon_{r}^{\prime }=1.71f^{1.13}+\frac{\varepsilon_{s}-4}{1+{\left(f/25\right)}^{2}}$$

(4) $$\sigma =1.35\sigma_{0.1}f^{0.13}+0.00222f^{2}\left[\frac{\varepsilon_{s}-4}{1+{\left(f/25\right)}^{2}}\right]$$

where f is the frequency, $\sigma_{0.1}=0.05$ and $\varepsilon_{s}=8.5$ were obtained based on the findings reported by Foster’s et al.

## 3. Microwave Breast Imaging 

Microwave imaging offers an accurate detection of breast tumors with specific reference to the shape, size and boundary irregularities [60]. During the past two decades, researchers around the world have investigated numerous of microwave imaging based approaches with particular focus on breast cancer detection and brain stroke detection. Microwave imaging approaches can be divided into two main groups: microwave tomography and radar-based imaging. 

### 3.1. Microwave Tomography

Microwave tomography offers quantitative information on dielectric properties of breast tissues to identify tumors [61]. Microwave tomography produces a map of permittivity and conductivity through inversion of those signals. However, the inverse problem takes much time due to the calculation process is complicated. Also, a nonlinear inverse scattering problem must be solved, and iterative image reconstruction algorithms are usually required to obtain a solution. In general, these ill-posed inverse scattering approaches suffer from non-uniqueness and require regularization in order to achieve convergence to a meaningful solution.

Several research groups have investigated microwave tomography breast imaging based on single frequency and multi-frequency approaches [62,63,64]. Among these groups, researchers from Dartmouth College has studied microwave tomography for breast cancer detection since 1990s, which is one of the representative research groups. They have developed a microwave imaging algorithm to map dielectric properties in a 2D lossy mediumin. The same research group has first developed a clinical prototype for imaging of human breasts. The prototype contains 32-channel data collection system at frequency range 500 MHz to 3 GHz. Clinical trial studies have shown that cm-size breast tumors could be identified using the multi-frequency microwave tomography technique [65]. However, microwave tomography requires heavy computation work that causes long image generate time. Recently, magnetic nanoparticles and compressive sensing techniques have been used in microwave tomography to represent as the contrast agent to improve the specificity, sensitivity and accuracy of the diagnosis of breast cancer [66,67,68].

### 3.2. Radar Based Microwave Imaging

Radar based microwave imaging is the main type of the microwave imaging-based approaches, which maps the internal organ structure by measuring the dielectric properties of the tissues [69]. Radar based microwave imaging has been proposed as a promising tool for early diagnosis of breast tumor with the advantages of cost-effective, safety, highly sensitive and specific. Additionally, it is a more comfortable and safer method compared to microwave tomography. Several experimental measurement systems were developed to demonstrate the radar-based microwave imaging approaches [70,71], including confocal microwave imaging (CMI) [72,73], microwave imaging via space time (MIST) [74,75] and holographic microwave imaging (HMI) [76,77]. 

A CMI was developed and evaluated for breast tumor detection [70]. The experimental results showed that the 2D CMI system can detect small tumors (2 mm in diameter) and the 3D CMI system can identify tumors with medium size (greater than 6 mm in diameter). In order to reduce artifacts and noises and enhance image, the research group has applied a delay multiply-and-sum method in CMI. However, the CMI method has not been validated on human subjects due to difficult implementation system. Recently, another major type of CMI method, namely tissue sensing adaptive radar (TSAR) imaging was proposed for detecting breast cancer [78]. Clinical trial studies indicated that the TSAR imaging method has the ability to detect breast lesions with correct size (greater than 4 mm in diameter) and location information. However, the TSAR method has the drawbacks of large reflections from skin and high-cost hardware system. The research team developed the Bayesian estimator to improve the image quality [79]. 

A MIST system that contains an array of 16 horn microwave sensors was developed for microwave breast imaging application [74], the proposed UWB horn sensor significantly improved the performance of breast cancer detection. However, artifacts were produced in the reconstructed images using MIST method. To improve the accuracy of the detection, the same research team has upgraded the measurement system. The experimental results demonstrated that the upgraded system can detect small tumors (4 mm in diameter). 

Elsdon et al. [80] proposed a near-field HMI for imaging of biological objects. Compared to other radar-based microwave imaging approaches, HMI has advantages in low-cost due to expensive ultra-high-speed electronics are not required as narrow-band signals can be converted to the baseband for digitization at a slower rate. However, the proposed near-field HMI technique only experimentally tested on a simple phantom. Different from Elsdon’s work, Wang et al. [81] recently developed a far-field HMI for breast tumor detection. The simulation and experimental results showed that various arbitrary shaped breast tumors with random sizes and locations can be clearly identified in the reconstructed breast images using the proposed single frequency HMI technique. The far-filed HMI method requires long data acquisition time, especially when generating 3D images. To solve this challenge, the authors recently applied compressive sensing technique in HMI to produce high-solution image using much less sampling rate. However, further experimental validations on realistic breast phantoms and human subjects are required in the future. 

## 4. RF Sensors for Biomedical Applications 

A radar-based microwave imaging system normally consists of a RF generator to illuminate microwave signals, RF sensor(s) to transmit microwave signals to the target object and measure the backscattered reflection signals from the object, and a computer with matched software (contains microwave imaging algorithm) to analysis the measured data to map the internal structure of the object. Microwave sensor plays an important role in the microwave breast imaging system. The image resolution can be improved by applying higher frequencies, develop a high sensitive sensor, and increase the number of the sensors applied in the system. In addition, smaller sensors enable a more number of sensors in the sensor array and enhance image resolution [82,83]. Various types of RF sensors have been developed for biomedical applications, which can be divided into the two main groups: microwave sensor for implementation of microwave breast imaging systems and microwave biosensor for cancer biomarkers detection.

### 4.1. Microwave Sensors for Microwave Breast Imaging Systems 

To date, numerous broadband and planar printed monopole microwave antennas have been developed for breast cancer detection due to their simple structure, broadband property, compact size, and ease-to-fabricate [84,85,86]. Recently, few flexible antennas were proposed to apply in the microwave sensor array for breast tumor detection [87,88,89,90]. Among these sensors, microstrip antenna is one of the most popular types of sensors developed for applying in microwave breast imaging systems because its compact size, inexpensive and can be printed directly onto a circuit board. A microstrip antenna normally contains a patch (metal foil) placed on the surface of the top board and a ground plane on the bottom side of the board, and the patch is normally made in different shapes such as square, rectangular, circular and elliptical [91]. 

In 2005, Shannon et al. [92] designed a slot line bow tie microwave antenna for identifying breast tumor. The antenna was immersed in a dielectric medium and fed with an integrated UWB balun. Both numerical and experimental validations were conducted to demonstrate the characteristics of the antenna on breast phantom with various cylindrical tumors. The obtained results showed that return loss of 10 dB is obtained in the frequency range of 2.5~9 GHz, and return loss of 5 dB is obtained in the frequency range of 1~10 GHz. Breast tumor (7 mm in diameter) at a depth of 4 cm from the aperture was successfully detected. 

In 2007, Nilavalan et al. [93] developed a low-profile stacked-patch antenna to radiate directly into a breast tissue model at frequency range of 4~9.5 GHz. This antenna produced a bandwidth of approximately 77% and beamwidths of approximately $\pm 40^{o}$ in the $\phi =0^{{}^{\circ}}$ plane and $\pm 30^{{}^{\circ}}$ in the $\phi =90^{{}^{\circ}}$ plane at 6.5 GHz. The antenna has been tested on breast phantom and experimental results showed that the proposed microstrip patch antenna has the potential to be applied in the microwave breast tumor detection system. 

In 2009, Shenouda et al. [94] proposed a dielectric-immersed antenna for a breast tumor detection system. The antenna had a tapered slot line that operates in a low permittivity dielectric. The sensor was fed with a microstrip-to-slot line balun at frequencies 2~12 GHz. Various phantoms were placed in a homogeneous to evaluate the suitability of the antenna for biomedical application. The antenna and balun were measured in canola oil, and good agreement between simulation and measurement was obtained. The −10 dB reflection coefficient bandwidth of the balun and antenna was obtained at frequencies 3~10.6 GHz. However, further validations on a more realistic 3D breast phantom were required in the future. 

In 2010, Bourqui et al. [95] developed a balanced antipodal Vivaldi antenna for applying in the TSAR system. The antenna made of three copper layers, the two external layers were connected to the feeding line ground planes and the central layer was connected to the signal conductor of the feeding line. The experimental results showed that the antenna provides better return loss above 2.4 GHz. The lower limit of the desired frequency bandwidth (2~12 GHz) was not reached, however the antenna still shows better than −7 dB reflection at that frequency. Gibbins et al. [96] developed a stacked-patch antenna and a wide-slot antenna for the purpose of breast tumor detection. The size of the wide-slot antenna was 3 times smaller than the stacked-patch antenna. The experimental results demonstrated that both antennas had suitable bandwidths for application in the UWB system and good agreement was found between simulation and experimental results for the wide-slot antenna. They also developed a hemispherical antenna array made of 16 stacked-patch antennas for breast tumor detection. Experimental results showed that the hemispherical antenna array can detect 8 mm spherical breast tumor phantoms at different locations.

In 2011, Wang et al. [97] designed a compact microstrip slot antenna for microwave breast imaging. The matching solution medium was required for the experimental performance. The antenna was experimentally validated on breast phantom and experimental results showed that it has achieved a good matching performance at 2~8 GHz. The antenna has the potential to be applied in a half-spherical antenna array for breast cancer detection, and it can also be adapted for other biomedical applications such as knee imaging.

In 2012, Chan et al. [98] designed and optimized a resistively loaded wire bowtie antenna based on a genetic algorithm approach and some empirical investigations. The antenna was experimentally tested in free space and within a tissue-like phantom. The impedance bandwidth of 100.75% was achieved with a VSWR < 2 at a frequency of 3.3~10.0 GHz. The experimental results agreed with the simulation results and both simulation and experimental results demonstrated that the proposed design has the potential for applications in microwave breast imaging prototype. In the same year, John et al. [99] developed an UWB bow tie sensor and a sensor array that made of 12 panels and each panel made of 3 UWB bow tie sensors for microwave breast imaging application. The coupling medium was filled in the cavity, and an image of a spherical object was reconstructed by using an inverse scattering algorithm. Bowtie sensor has benefits in compact, wideband and easy-to-manufacture. 

In 2013, Wang et al. [100] developed an open-ended waveguide sensor for application in an HMI system for detecting breast tumor. The spiral sensor array and random sensor array were proposed and compared with the regular spaced sensor array. Each sensor array was made of 16 open-ended waveguide sensors with one worked as the transmitter and the others worked as the receivers. The sensors offered good performance in the frequency range of 10~20 GHz. Both simulation and experimental results demonstrate that the proposed waveguide antenna has the ability to identify small breast tumors located at breast phantoms. The breast phantom image quality was significantly improved by using spiral and random sensor arrays. 

In 2014, Nepote et al. [101] proposed a horn antenna for breast radar imaging applications in the frequency range of 1.5~6 GHz. The designed horn antenna was experimentally evaluated on various breast phantoms and the results were compared with the Vivaldi sensor. Images obtained using the horn antenna had a lower noise level and higher contrast than the images obtained using the Vivaldi antenna. In the same year, Ahadi [102] developed a square monopole antenna to identify breast tumor. Effect of variation in different parts of the antenna was analyzed and presented to optimize the antenna for its best operation. The design was numerically and experimentally validated to demonstrate its characteristics. The results showed that the antenna has <10 dB feed match at frequency 4 GHz to more than 9 GHz. It has been represented that in order to minimize the distortion in the transmitted signal through the breast the antenna S21 is about 5 dB at a frequency range of 4~8 GHz, which is suitable for the microwave imaging application. 

In 2015, Kahar et al. [103] proposed an UWB microstrip monopole antenna for imaging of heterogeneous breast model. A heterogeneous breast model was developed to validate the proposed antenna with different locations from skin and tumor. Simulation results showed that the antenna has high gain, phase linearity, and good polarization characteristics. High current density was observed in the most deep-seated tumor as well as for the smallest tumor, keeping SAR values on breast tissues well within safe limits. The best simulation results were achieved when the antenna was placed at 1 mm away from skin. Bahramiabarghouei et al. [87] developed a single microstrip sensor array and a dual polarization microstrip sensor array for radar-based imaging application. The sensor arrays made of 16 flexible monopole antennas and 16 flexible spiral antennas, respectively. The operating frequency range was 2~4 GHz for both arrays. Experimental results showed that the developed flexible antennas have good impedance matching when in different positions with different curvature around the breast. By using a reflector for the arrays, the penetration of the propagated electromagnetic waves from the antennas into the breast can be improved by factors of 3.3 and 2.6, respectively.

In 2016, Karli et al. [104] developed a compact microstrip antenna for implementation in an UWB microwave imaging system. The design was numerically and experimentally tested on various breast phantoms for identifying breast tumors. The experimental results demonstrated that the proposed antenna has sufficient sensitivity and effectiveness to detect tumors when the antenna is in contact with the breast skin. Such design may enhance the accurate detection of breast tumors when it is applied in the imaging measurement system. Li et al. [105] designed a circularly polarized implantable patch antenna for industrial, scientific, and biomedical applications. The proposed antenna can obtain improvement for both impedance bandwidth and axial ratio bandwidth, without increasing the backward radiation. The proposed antenna has the potential for biomedical applications at 2.45 GHz. In the same year, a cost-effective wearable bra was developed for microwave breast cancer detection [89]. The bra contains an array of 16 flexible microwave sensors, which is highly cost-effective compared to typical table-based microwave imaging systems. The developed wearable prototype was tested on healthy volunteers. Experimental results showed that the proposed wearable bra offers better performance than the table-based microwave imaging system. However, only one healthy volunteer was involved in this study, further experimental validations on a wider range of human subjects with varying breast size and density are required in the future. 

**Figure 1:**
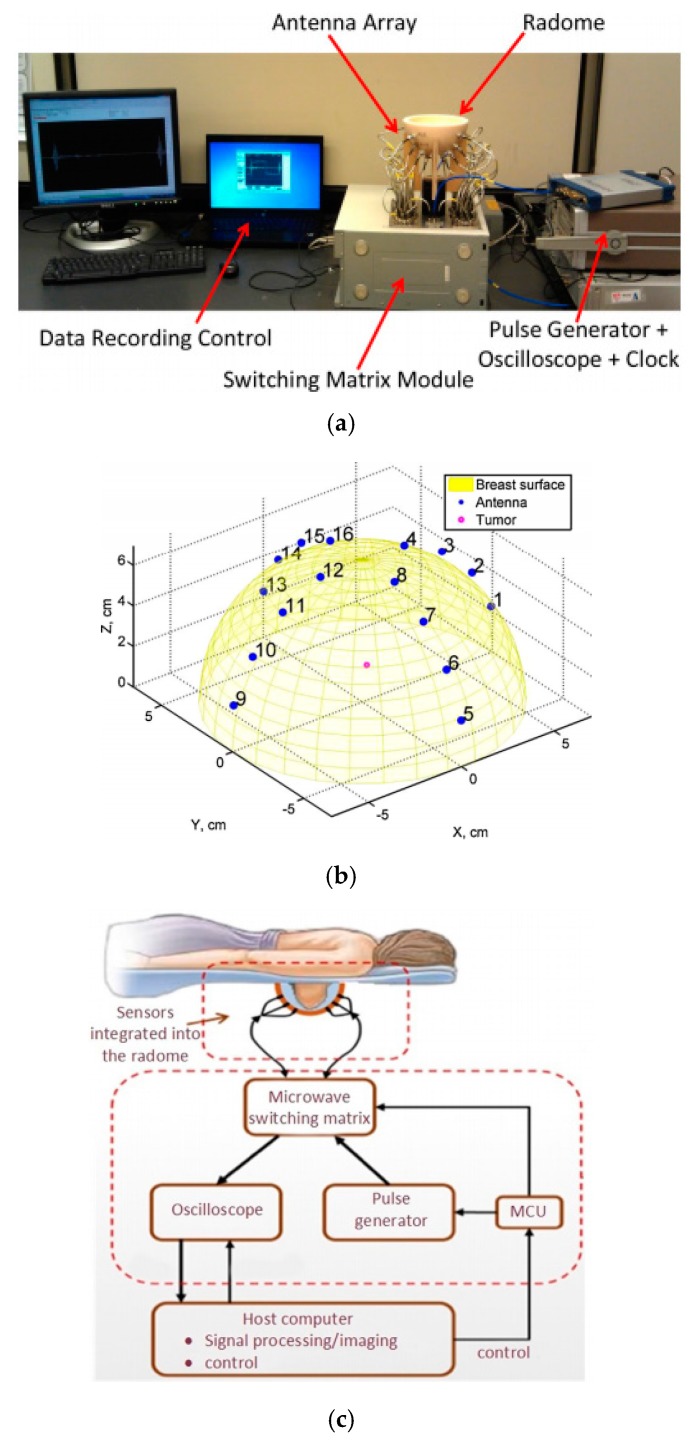
(**a**) Microwave imaging measurement system developed by Li et al.; (**b**) Microwave sensor array configuration; (**c**) Schematic diagram of experiment. Reprinted with copyright permission from Li et al. [106].

In 2017, Li et al. [106] applied cost-sensitive ensemble classifiers to the microwave imaging system (see Figure 1) to identify abnormalities in the breast. A hemispherical ceramic dielectric radome was designed for performing breast scans on subjects, which houses the target breast and the 16-element microwave sensor array. A gel (such as ultrasound gel) or liquid was filled in the space between the skin and the randome walls, due to the fact that the radome was designed for a largest breast size. During data collection, a Gaussian-modulated pulse wave was generated and shaped by a passive microwave filter in the frequency range of 2~4 GHz. The transmitted and reflected signals from the breast were measured and recorded by all microwave sensors located in the sensor array. The total of 240 signals were recorded from 16 sensors per scan with less than 2 minutes. The proposed cost-sensitive ensemble classification techniques were evaluated with measurements from breast phantoms and patients using their developed microwave screening system. Experimental results showed that the ensemble selection-based algorithm significantly outperforms other detection techniques for the clinical trial data set. However, only healthy patients were involved in this study. 

**Figure 2:**
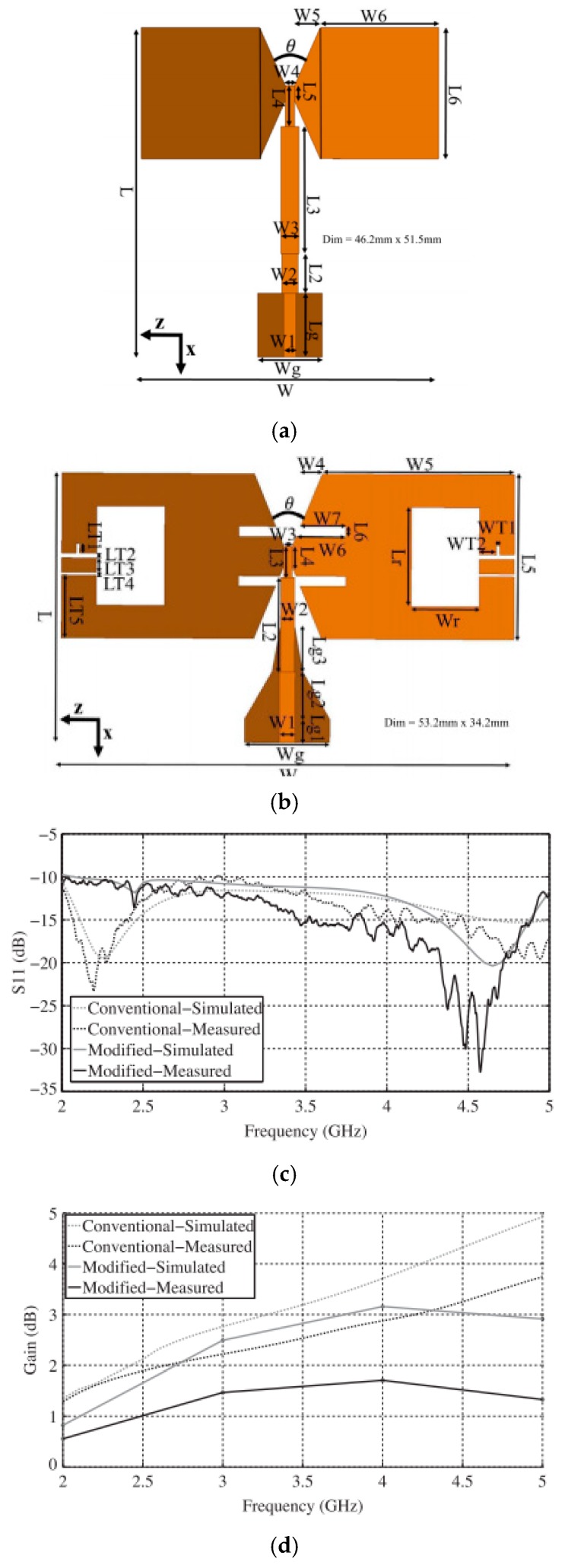
(**a**) Conventional antenna geometry; (**b**) Modified bow-tie antenna; (**c**) Simulated and measured return loss; (**d**) Simulated and measured gain. Reprinted with copyright permission from Ting et al. [107].

Recently, Ting et al. [107] developed a bow-tie antenna with low cross-polarization level and miniaturization (Figure 2). The antenna was fabricated on RO4003 substrate and experimental validation was conducted to demonstrate the characteristics include return loss, gain and radiation pattern. The comparison study between the proposed antenna and the conventional bowtie antenna was also conducted. The experimental results demonstrated that the new proposed antenna offers a cross-polarization improvement over $\pm 120^{{}^{\circ}}$ around the boresight at frequency range of 2~5 GHz. The new proposed antenna has the potential for biomedical application. 

### 4.2. RF Biosensors for Cancer Biomarker Detection

RF biosensor offers a promising new approach for accurate, safe, label-free, and rapid diagnosis of biomolecules and cancer cells. Compared to RF sensors, RF biosensor offers low-cost, disposable, and high-sensitive option for biomolecule diagnostic systems. RF biosensors can be classified as near-field biosensors and far-field biosensors. The section mainly addresses near-field RF biosensors. Microwave components, such as transmission lines, lumped capacitors, waveguides, fabricated on a substrate and used for detection of biological materials, can be termed as passive sensors [108]. The design of RF biosensor requires to meet the specific design requirements, such as working frequency, bandwidth, directivity, sensitivity, accuracy, compact size and low cost. Various nanomaterials have been applied to develop RF biosensors in order to enhance the sensitivity of biomolecule detection. 

Lee et al. [109] developed a planar split-ring resonator-based microwave biosensor for label-free detection of biomolecules such as prostate cancer marker, prostate specific antigen (PSA), and cortisol stress hormone. The biosensor consisted of a resonance-assisted transducer and was excited by a time-varying magnetic field component of a local high-impedance microstrip line. The device exhibits an intrinsic S21 resonance with a quality-factor of 50. For the Biomolecular interaction, anti-PSA and anticortisol were immobilized on the gold surface of the resonator by a protein-G mediated bioconjugation process and corresponding frequency shifts of 30 ± 2 MHz (for anti-PSA) and 20 ± 3 MHz (for anti-cortisol) were observed.

Yang et al. [110] developed a multilayered polymeric DNA biosensor using RF technology with gold and magnetic nanoparticles to enhance the detection sensitivity of DNA. Previous studies have shown that the nuclear magnetic resonance-based RF biosensor has an ability to detect various biomolecules such as avidin, human chorionic gonadotropin, and human bladder cancer cells. Kim et al. [111] developed a wireless RF biosensor to demonstrate the biomolecular binding systems such as biotin–streptavidin and DNA hybridization. Chen et al. [31] proposed a microwave biosensor dedicated to the dielectric spectroscopy of a single and living biological cell in its liquid culture medium in the micro and millimeter wave ranges. The sensor worked in the near field and involves a capacitive gap to perform the electromagnetic sensing, while a microfluidic system was developed and adapted to the RF circuit to precisely localize the single biological cell under study. Both capacitive and conductive contrasts of a living biological cell measured in its culture medium were accessible. A living B lymphoma cell was measured in the frequency range of 40 MHz~40 GHz, with a measured capacitive contrast of the order of several hundreds of attofarads.

Camli et al. [112] designed a simple and cost-effective microwave biosensor based on microstrip antenna driven ring resonator for label-free detection of glucose. Simulation and experimental validations were conducted to demonstrate the sensing capacity for changes in dielectric properties of the surrounding medium. The simulation and experimental results were in good agreement. Recently, Garrett et al. [113] reported the significant progress made on the average dielectric property analysis of complex breast tissue with microwave transmission measurements. More recently, Tselev et al. [114] applied the microwave microscopy for in situ imaging of live biological cells to identify changes in malignant tissues.

## 5. Challenges and Future Works

Microwave imaging has been recently proposed as an alternative or additional approach to the current standard X-ray mammography for early breast cancer detection. Apart from microwave imaging algorithms, microwave sensors and sensor arrays have been reported play the most important role in the microwave imaging systems for diagnosis of breast tumors. Some major limitations have been reported for practical implementation of microwave imaging-based methods, including low dielectric property contrasts between the healthy and the malignant tissues, working frequency selection, development of a high sensitivity microwave sensor, and limited image resolution. Previous studies have suggested that it is necessary to develop a high sensitivity microwave sensor and a sensor array to improve the image resolution and reduce the system cost. Many investigators have increased the number of implementation sensors in a microwave imaging sensor array to improve the image quality, for example, Kurrant et al. [115] increased the number of microwave sensors in the measurement system from 16 to 256. However, this increment may reduce the accuracy of tumor detection due to the mutual coupling signals caused between sensors. Moreover, the total operating cost of the system will be increased and the measurement system will be becoming more complex with increasing the number of sensors.

To address these challenges, investigators recommended that more investigations should be provided on the development of a high dynamic measurement system with particular focus on high sensitive, compact and low-cost microwave sensors and sensor arrays to achieve high quality images. In recent years, many researchers have developed a numerous of high sensitive RF sensors for application in the microwave imaging system for detecting breast tumors. Most of these sensors have been extensively tested on various simplified breast phantoms both numerically and experimentally. Coupling solution medium was filled in the space between the target object and the sensors in most of microwave imaging systems in order to reduce the noise and improve the image resolution. However, such method also increases the operating cost significantly. Optimization of sensor arrays such as using unequally spaced sensor arrays and applying compressed sensing approaches in the signal and image processing may be other solutions to improve the image quality in a fast and cost-effective manner. 

In recent years, biosensors and biomarkers-based techniques for early breast cancer detection have attacked many people’s interests. To date, cancer biomarker discovery is still in its discovery stage and the evidence is too restricted to confidently apply biomarkers as diagnostic tools for diagnosing early-stage breast cancer. Protein biomarkers have utility within a panel of biomarkers, however, they have not been recommended as individual biomarkers to detect breast cancer. Biosensor techniques have some important drawbacks that are related to the integration of the diagnosis of breast cancer in primary health care. For instance, QCM-based biosensors are more common and reliable platforms than other types of sensors for surgery applications. However, there are some drawbacks of biosensors such as small target size, marker levels, the possibility of high non-specific binding in the case of serum or real patient samples. Recent research trends of nano-biosensors and RF biosensors for biomolecules offer great potential for early cancer detection. However, these techniques are not mature for clinical trials. Future investigations should be addressed directly to improve the selectivity, sensitivity, accuracy, and multiplexing capacity of microwave sensors.

## 6. Conclusions

Successful clinical trials of microwave breast imaging demonstrated that microwave imaging has the potential to become an additional or alternative method to the current standard X-ray mammography for detecting breast tumors in their early stages. Microwave sensor plays the most important role in the microwave imaging measurement systems. This paper presented an exhaustive summary of microwave sensors for applications in microwave imaging approaches for breast tumor detection, including electric properties of biological tissues, microwave imaging methods, microwave sensors and microwave biosensors. Microwave images of breast have direct impacts on spatial resolution, microwave sensors and sensor arrays, optical choice of frequency, detection accuracy and quality of imaging. Several advantages of existing microwave sensors, open challenges, possible solutions and future work directions also discussed.
